# Pigment epithelium-derived factor mediates retinal ganglion cell neuroprotection by suppression of caspase-2

**DOI:** 10.1038/s41419-019-1379-6

**Published:** 2019-02-04

**Authors:** Vasanthy Vigneswara, Zubair Ahmed

**Affiliations:** Neuroscience and Ophthalmology, Institute of Inflammation and Ageing, College of Medical and Dental Sciences, University of Birmingham, Birmingham, B15 2TT UK

## Abstract

Retinal ganglion cells (RGCs) undergo rapid cell death by apoptosis after injury but can be rescued by suppression of caspase-2 (CASP2) using an siRNA to CASP2 (siCASP2). Pigment epithelium-derived factor (PEDF), has neuroprotective and anti-angiogenic functions and protects RGC from death. The purpose of this study was to investigate if suppression of CASP2 is a possible mechanism of neuroprotection by PEDF in RGC. Adult rat retinal cells were treated in vitro with sub-optimal and optimal concentrations of siCASP2 and PEDF and levels of CASP2 mRNA and RGC survival were then quantified. Optic nerve crush (ONC) injury followed by intravitreal injections of siCASP2 or PEDF and eye drops of PEDF-34 were also used to determine CASP2 mRNA and protein reduction. Results showed that PEDF and PEDF-34 significantly suppressed CASP2 mRNA in culture, by 1.85- and 3.04-fold, respectively, and increased RGC survival by 63.2 ± 3.8% and 81.9 ± 6.6%, respectively compared to cells grown in Neurobasal-A alone. RGC survival was significantly reduced in glial proliferation inhibited and purified RGC cultures suggesting that some of the effects of PEDF were glia-mediated. In addition, intravitreal injection of PEDF and eye drops of PEDF-34 after ONC also suppressed CASP2 mRNA levels by 1.82- and 3.89-fold and cleaved caspase-2 (C-CASP2) protein levels by 4.98- and 8.93-fold compared to ONC + PBS vehicle groups, respectively, without affecting other executioner caspases. Treatment of retinal cultures with PEDF and PEDF-34 promoted the secretion of neurotrophic factors (NTF) into the culture media, of which brain-derived neurotrophic factor (BDNF) caused the greatest reduction in CASP2 mRNA and C-CASP2 protein. The neuroprotective effects of PEDF were blocked by a polyclonal antibody and PEDF suppressed key elements in the apoptotic pathway. In conclusion, this study shows that some of the RGC neuroprotective effects of PEDF is regulated through suppression of CASP2 and downstream apoptotic signalling molecules.

## Introduction

Optic nerve crush (ONC) leads to the rapid death of retinal ganglion cells (RGCs) by apoptosis, attributed to the loss of trophic support. However, RGC can be protected from death by a variety of neurotrophic factors (NTFs) including brain-derived neurotrophic factor, ciliary neurotrophic factor and glial cell line-derived neurotrophic factor^1–4^. Others and our recent work have shown that pigment epithelium-derived factor (PEDF), a 50 kDa neurotrophic factor belonging to the serpin superfamily, is neuroprotective for a wide variety of CNS neurons including RGC^5–12^. Furthermore, PEDF promotes significant RGC axon regeneration after ONC and a neurotrophic fragment of PEDF and PEDF-34, penetrates into the vitreous body and retina after topical delivery and promotes significant RGC survival and axon regeneration^12^.

Caspases are a family of cysteine-rich proteases that are expressed as pro-caspases and activated either by proximity-induced dimerisation or proteolytic cleavage and orchestrate apoptosis during development and in the adult^13^. Once activated, caspases target regulatory proteins involved in DNA replication, DNA repair, cell survival signalling and the cytoskeletal reorganisation^14–19^. Caspase-2 (CASP2) is the most highly conserved of the caspases and is specifically activated in RGC after ONC^20–23^. Inhibition of CASP2 with a stable modified siRNA to CASP2 (siCASP2) promoted the survival of >95% of RGC from death for up to 12 weeks after ONC^20–24^. These studies demonstrate that CASP2 activation is required for RGC death.

CASP2 resembles other caspase in possessing a long pro-domain containing a caspase recruitment domain (CARD) sequence and is activated by proximity-induced dimerisation which is facilitated by various adaptor molecules^13^. For CASP2, the adapter protein receptor interacting protein-associated interleukin-1β-converting enzyme/CED-3 homologue 1 protein with a death domain (RAIDD), interacts with CARD via its death domain (DD) and activates CASP2^25^. Later studies showed that RAIDD can also interact with p53-inducible protein with a death domain (PIDD) via the DD and hence forming the PIDDosome, composed of PIDD, RAIDD, and C-CASP2 as the activation complex for CASP2^26^. More recently, RAIDD and not PIDD was shown to be required for CASP2-dependent hippocampal neuronal death, activated by either NGF withdrawal or Aβ_1-42_ peptide treatment^27^. Moreover, activation of CASP2 promoted the induction of downstream Bcl-2-interacting mediator of death (Bim) and that this was mediated by CASP2-dependent activation of the transcription factor, c-Jun^28^.

In this study, we evaluated, in vitro and in vivo, the levels of CASP2 expression in RGC after treatment with PEDF and PEDF-34 to determine how PEDF and PEDF-34 exerts its neuroprotective effect on RGC. We further investigated whether PEDF treatment modulated RAIDD or PIDD or downstream c-Jun and Bim in the pathway to activation of CASP2 after ONC in RGC death.

## Materials and methods

### Experimental design: in vitro experiments

For all in vitro experiments, retinal cells were cultured from 6–8-week-old adult female Sprague Dawley rats (170–220 g)^11^ and experimental conditions for PEDF and PEDF-34 treatment included retinal cells treated with: (1), supplemented Neurobasal-A (NBA; supplemented with B27 supplement and l-Glutamine; all from Invitrogen, Paisley, UK) alone; and previously optimised doses of (2), 2.5 × 10^–5^µg/µL human PEDF (equivalent to 463 pM; Phoenix Europe GmbH, Karlsruhe, Germany); (3), 5.4 pM human PEDF-34 (amino acids 44–77 of the N-terminus of human PEDF protein (PFFKVPVNKLAAAVSNFGYDLYRVRSSTSPTTNV) chemically synthesised by Vigneswara et al.^11^) and (4), 5.4 pM human PEDF-44 (amino acids 78–101; Phoenix Europe GmbH)^11,12^.

For knockdown of CASP2 mRNA, retinal cells were treated with: (1), NBA alone; (2), Lipofectamine 2000 alone (Lipofectamine); (3), pre-optimised non-specific siGFP (50 nM); (4) pre-optimised siCNL, a control scrambled version of siCASP2 (50 nM); and (5), varying concentrations of siCASP2 (10–200 nM^20^).

All in vitro experiments described in this manuscript were performed in triplicate wells and repeated on three independent occasions (total *n* = 9 wells/treatment) by an investigator masked to the treatment conditions.

### Mixed adult retinal cultures

Mixed adult rat retinal cultures containing enriched populations of RGC were prepared as described previously^11^. Briefly, retinal cells were dissociated using a Papain dissociation kit according to the manufacturer’s instructions (Worthington Biochemicals, Lakewood, NJ, USA). 125 × 10^3^ retinal cells/well were plated in poly-d-lysine and laminin pre-coated 8-well chamber slides and cultured in NBA. Cells were cultured for 4 days (d) at 37^o^C and 5% CO_2_ before RNA extraction for qRT-PCR or fixation in 4% formaldehyde for immunohistochemistry.

To limit glial cell proliferation, mixed retinal cultures were treated with 30 µM 5-fluro-2-deoxyuridine (5-FDU, Sigma)^29^.

### Purified adult RGC cultures

RGCs were purified from twenty 6–8-week-old adult Sprague-Dawley rat retinae and purified by sequential immunopanning, as described for postnatal rats^30^. Briefly, retinae were dissociated using neural tissue dissociation kit containing papain and sequential passaging through serological and fire-polished Pasteur pipettes according to the manufacturer’s instructions (Miltenyi Biotec, Woking, UK). Two culture dishes were used for depletion and were coated in anti-rabbit IgM and goat anti-rabbit IgM followed by anti-rat CD90.1/Thy1.1 antibody (Cat No. ab203022; Abcam, Cambridge, UK). Cells were allowed to adhere to the first depletion plate and the non-adherent cells were subjected to a further round of depletion before being applied to the second selection plate. Cells adhering to the second plate were washed, collected by trypsinization and subsequent centrifugation before being plated in 8-well chamber slides at a plating density of 10,000 RGC/well in supplemented NBA and incubated at 37^o^C and 5% CO_2_. Cells were cultured for 4 days prior to harvesting and total RNA extraction for qRT-PCR.

### Knockdown of CASP2

For the siCASP2 knockdown experiments in mixed adult retinal cells, the optimal dose of siCASP2 to knock down CASP2 mRNA was determined by qRT-PCR in a preliminary experiment using pre-optimised CASP2 primers^20^. An initial dose–response curve with 5, 10, 20, 50, 100 and 200 nM of siCASP2 was used to determine that the optimal dose of siCASP2 was 50 nM, which caused 95% knockdown of CASP2 mRNA. Controls for this experiment included control cells incubated in NBA alone, Lipofectamine 2000, pre-optimised non-specific siGFP and siCNL^20^.

Other rat CASP2 siRNA sequences were pre-optimised for CASP2 mRNA knockdown in retinal cultures prior to use (data not shown) and included: 20 nM S1–5′-GCCAUGCACUCCUGAGUUU-3′;^28^ 20 nM S2 – CASP2 siRNA, cat no. SC-72108 (Santa Cruz Biotechnology, San Diego, CA, USA); and 10 nM S3 – CASP2 siRNA, cat no. AM16708 (Assay ID 194613) (ThermoFisher Scientific, Loughborough, UK), which gave maximum knockdown efficiencies of 70% in mixed and purified retinal cultures.

Mixed retinal and purified adult rat RGC cultures were treated with the optimised concentrations of siCASP2, PEDF, PEDF-34 and PEDF-44 and appropriate controls as described for mixed retinal cells above. All experiments were performed in triplicate and repeated on three independent occasions.

### Immunocytochemistry of retinal cultures

Fixed cells were washed in several changes of phosphate buffered saline (PBS) before permeablisation and blocking in PBS containing 3% bovine serum albumin (BSA) and 0.1% Triton X-100 (both from Sigma, Poole, UK). Cells were then incubated with monoclonal anti-βIII-tubulin antibody (Cat No. T8660; 1:200 dilution; Sigma) for 1 h at room temperature (RT) and used to localise RGC and their neurites. Cells were then washed in several changes of PBS and incubated with Alexa 488 anti-mouse IgG (Cat No. A-11001; 1:400 dilution; Invitrogen) for 1 h at RT, washed in several changes of PBS and mounted using Vectashield containing DAPI (Vector Laboratories, Peterborough, UK). Slides were then anonymised by a second investigator and viewed using a Zeiss epi-fluorescent microscope equipped with an AxioCam HRc and Axiovision Image capture software (all from Zeiss, Hertfordshire, UK). Immunocytochemistry included controls with primary antibody omitted, that were used to set background levels of nonspecific staining (not shown) prior to image capture.

### Quantification of RGC survival

Each well of the chamber slides were divided into nine quadrants and images captured randomly from each quadrant. RGC survival was then determined by counting the number of surviving βIII-tubulin^+^ RGC in each of the 9 quadrants/well and total RGC survival determined through multiplying by the area of each chamber.

### Quantification of non-neuronal cells

Non-neuronal cells were counted in 8-well chamber slides as described by us previously^31^. Briefly, each 8-well chamber slide was partitioned into nine quadrants and DAPI counterstained cells (all cells) were subtracted from those stained with βIII-tubulin (RGC) to give an estimate of the number of neuronal cells versus non-neuronal cells.

### Assessment of neurotrophic factor levels in culture medium by ELISA

Mixed adult rat retinal cultures containing enriched populations of RGC were prepared as described in 2.2. above. Retinal cells were treated with 5.4 pM human PEDF-34 (Peprotech) in NBA for 5 h before carefully washing cells in 3 changes of PBS to remove all traces of PEDF-34. NBA was then replaced into each well and cells incubated at 37^o^C and 5% CO_2_ for 3 days. To limit glial cell proliferation, cultures were also treated with 30 µM 5-FDU (Sigma) for the duration of the experiment^29^. Culture media was collected, centrifuged to remove cell debris and subjected to ELISA to detect NTFs: brain-derived neurotrophic factor (BDNF); ciliary neurotrophic factor (CNTF); glial cell line-derived factor (GDNF); nerve growth factor (NGF) and neurotrophin-3 (NT-3) (all from Abcam), according to the manufacturer’s instructions.

### PEDF antibody blocking experiments

In a preliminary dose response experiment, the optimal concentration of goat polyclonal anti-rat PEDF antibody (Cat No. AF1177; R&D Systems Europe) to antagonise the effects of pre-optimised PEDF and PEDF-34 on RGC survival in vitro was determined^11,12^. PEDF (563 pM) and PEDF-34 (5.4 pM) was mixed with varying amounts of PEDF antibody ranging from 1 to 50 µg/ml dissolved in PBS for 2 h at RT before adding onto cultured retinal cells and incubated at 37 ^o^C and 5% CO_2_ for 3 days. Control wells included PEDF and PEDF-34 that were pre-treated with an equal volume of PBS (vehicle) or an equal concentration of normal rat IgG control (non-specific antibody control). Cells were then subjected to immunocytochemistry for βIII-tubulin and determination of βIII-tubulin^+^ RGC survival as described above. We determined that 15 µg/ml was sufficient to completely block the neuroprotective effect of PEDF and PEDF-34 whilst neither vehicle nor control rat IgG-treated wells affected RGC survival (not shown).

In further experiments, mixed adult retinal cultures were subjected to pre-optimised PEDF blocking antibodies and used to determine changes in CASP2 mRNA and protein and the neuroprotective effects on RGC survival determined in the presence or absence of PEDF blocking antibody.

### Experimental Design: in vivo experiments

All in vivo tissue samples used for western blot and immunohistochemistry were derived from samples collected as part of previously published studies^11,12,20,22,23^. Animal procedures were licenced by the UK Home Office and approved by the University of Birmingham’s Ethical Review Board. All animal surgeries were carried out in strict accordance to the guidelines of the UK Animals Scientific Procedures Act, 1986 and the Revised European Directive 1010/63/EU and conformed to the guidelines and recommendation of the use of animals by the Federation of the European Laboratory Animal Science Associations. Experiments also conformed to the ARVO statement for use of animals in research, except that bilateral optic nerve crushes are a condition of our licence, imposed by the UK Home Office. This is viewed as ‘reduction’ in keeping with the 3 R’s principle, since rats do not rely on sight as a primary sense and none of the normal behaviours are altered as a result. Adult female Sprague-Dawley rats (6–8-week-old, 170–220 g) were used for all in vivo experiments and randomly assigned to each treatment group presented in this study comprising 6 eyes (*n* = 6 retinae/3 rats) and repeated on three separate occasions (total = 18 eyes (*n* = 18 retinae/9 rats)/treatment group)^11,12^. Briefly, optic nerves were crushed bilaterally 2 mm from the globe in anaesthetised rats as described previously^32^. PEDF or PEDF-34 were either injected intravitreally every 8 days after optic nerve crush (ONC) until the end of the experiment or given as topical eye drops every day, respectively, based on previously optimised dose frequency/concentration studies^11,12^.

Animals were allowed to survive for up to 28d prior to killing by CO_2_ overdose and retinae were either snap frozen in liquid nitrogen (for RNA (*n* = 9 rats (18 retinae)/group from three independent repeats; 63 rats in total) and total protein extraction (*n* = 9 rats (18 retinae)/group from three independent repeats; 63 rats in total) for qRT-PCR and Western blot) or whole eyes fixed in 4% paraformaldehyde (*n* = 9 rats (18 retinae)/group from three independent repeats; 27 rats in total (3 groups)) for cryoprotection and immunohistochemistry. To determine the effect of PEDF-34 eye drops over 21d on caspase-2 levels by qRT-PCR, RNA was extracted from 6 rats (12 retinae)/group from two independent repeats; a total of 54 rats. To determine changes in C-CASP2 levels over 3d by western blot and RAIDD, PIDD, phospho-c-Jun, c-Jun and Bim levels by western blot at 3d, 9 rats (18 retinae)/group from three independent repeats were used to extract total protein; a total of 54 rats. To analyse mRNA and protein levels by qRT-PCR and western blot, each independent repeat comprising 6 eyes (3 rats)/treatment were pooled, treated and analysed separately prior to averaging the data over the two or three independent repeats to achieve the final data presented in the figures of this study. All rats were included in the study as there were no unsuccessful ONC animals and no rats suffered complications during surgery.

### Preparation of PEDF, PEDF-34 and PEDF-44 for in vivo use

PEDF was dissolved in PBS and 1.88 nM final concentrations in 5 μl were intravitreally injected into appropriate eyes under anaesthesia and as previously pre-optimised in vivo^11^.

Eye drops of PEDF-34/44 were prepared fresh daily in 0.9% saline and 1.88 nM final concentrations in a volume of 5 µl were applied topically as previously pre-optimised in vivo^12^. Briefly, animals were lightly anesthetised to prevent blinking and 5 μl of PEDF was dropped onto the surface of the eye and repeated daily for 28d.

### Tissue preparation and sectioning

Fixed whole eyes were post-fixed for 2 h in 4% paraformaldehyde, cryoprotected through a graded sucrose solution, blocked up in OCT embedding compound (Raymond A Lamb, Eastbourne, UK), cut at 15-μm-thick using a cryostat (Brights Instruments, Huntingdon, UK) and adhered onto charged slides prior to immunohistochemistry, as described previously^12^.

### Immunohistochemistry of retinal sections

Sections were washed in PBS, permeablised in 0.1% Triton X-100 (Sigma) in PBS and nonspecific binding blocked in 3% bovine serum albumin (BSA) in PBS for 30 min. Appropriate primary antibodies (Table 1) were diluted in PBS containing 3% BSA and 0.05% Tween 20 and incubated overnight in a humidified chamber at 4^o^C. Sections were then washed in PBS and incubated with corresponding Alexa488 and Texas Red labelled anti-mouse and anti-rabbit IgG, diluted in PBS containing BSA and 0.05% Tween 20 and incubated in a humidified chamber for 1 h at room temperature. Sections were then washed in PBS and coverslips mounted using Vectashield containing DAPI (Vector Laboratories), examined using an Axioplan-2 epi-fluorescent microscope and images captured using an AxioCam HRc as described above. Immunocytochemistry included controls with primary antibody omitted, that were used to set background levels of nonspecific staining (not shown) prior to image capture.

**Table 1:** List of primary and secondary antibodies used for western blot (WB), immunohistochemistry (IHC) and immunocytochemistry (ICC)

Antibody	Source	Cat No.	Dilution	
*Primary antibodies*				
			WB	IHC/ICC
Goat anti-cleaved caspase-2 (p12)	Santa Cruz Biotechnology, San Diego, CA, USA	sc-623	1:200	1:200
Rabbit anti-cleaved caspase-3	Cell Signalling Technology, Danvers, MA, USA	9664	1:200	
Rabbit anti-cleaved caspase-6	Cell Signalling Technology, Danvers, MA, USA	9761	1:200	
Rabbit anti-cleaved caspase-7	Cell Signalling Technology, Danvers, MA, USA	8438	1:200	
Rabbit anti-cleaved caspase-8	Cell Signalling Technology, Danvers, MA, USA	9496	1:200	
Rabbit anti-RAIDD	Abcam, Cambridge, UK	ab76465	1:1000	
Rabbit anti-PIDD	Merck Millipore, Watford, UK	MACB189	1:1000	
Rabbit anti-Phospho-c-Jun^(Ser63)^	Cell Signalling Technology, Danvers, MA, USA	2361	1:750	
Rabbit anti-c-Jun	Cell Signalling Technology, Danvers, MA, USA	9165	1:750	
Rabbit anti-Bim	Cell Signalling Technology, Danvers, MA, USA	2933	1:1000	
Mouse anti-βIII tubulin	Sigma, Poole, UK	T8660		1:500
Mouse anti-β actin	Sigma, Poole, UK	A5441		1:400
*Secondary antibodies*				
HRP-labelled anti-mouse IgG	GE Healthcare, Buckingham, UK	NA931	1:1000	
HRP-labelled anti-rabbit IgG	GE Healthcare, Buckingham, UK	NA934	1:1000	
HRP-labelled anti-goat IgG	Santa Cruz Biotechnology, San Diego, CA, USA	sc-2020	1:1000	
Alexa594 anti-goat IgG	Invitrogen, Poole, UK	A-11058		1:400
Alexa488 anti-mouse IgG	Invitrogen, Poole, UK	A-11001		1:400
Alexa488 anti-rabbit IgG	Invitrogen, Poole, UK	A-21206		1:400

### RNA extraction and qRT-PCR

Total RNA from cells and eye tissues were extracted using Trizol reagent (Invitrogen) according to the manufacturer’s instructions, reverse transcribed into cDNA and the levels of rat CASP2 (5′-GGCGGAGGAGTTCCACATTC-3′ (forward) and 5′-GTGCAGGGTCCGAGGT-3′ (reverse)), RAIDD (Cat no. 4331182, Rn0109149-m1, ThermoFisher), PIDD (Cat no. 4351372, Rn01479823_g1, ThermoFisher), Bim (Cat no. 4331182, Rn00674175_m1) mRNA was detected by qRT-PCR, as described by us previously^20^. Briefly, qPCR was performed using a Bio-Rad iQ5 PCR detection system with SYBR green PCR mastermix (Thermo Fisher Scientific, Paisley, UK). Primer efficiency was tested using a 7-point standard curve, and gene expression calculated using the 2−ΔΔCt method and normalised to the expression of β-actin (5′-ACCTTCTACAATGAGCTGCG-3′ (forward) and 5′-CTGGAATGGCTACGTACATGG-3′ (reverse).

### Protein extraction and western blot analysis

Protein extraction and western blot was performed according to our previously published studies^31^. Briefly, retinal cultures in vitro or retinal tissue from in vivo experiments were lysed with ice-cold lysis buffer and lysates collected and clarified by centrifugation. 20 μg of total protein extract was resolved on 10% SDS gels, transferred to polyvinylidine fluoride membranes and probed with relevant primary antibodies as detailed in Table 1, followed by incubation with appropriate HRP-labelled anti-rabbit or anti-mouse IgG (GE Healthcare, Buckinghamshire, UK). Bands were detected using the enhanced chemiluminescence kit (GE Healthcare). Mouse anti β-actin primary antibody, followed by HRP-labelled anti-mouse IgG (GE Healthcare) was used as a protein loading control.

### Densitometry

Western blots were scanned into Adobe Photoshop (Adobe Systems Inc, San Jose, CA, USA) keeping all scanning parameters the same between blots. The integrated density of bands were then analysed using the built-in-macros for gel analysis in ImageJ (NIH, USA, http://imagej.nih.gov/ij) and means ± SEM were plotted in Microsoft Excel (Microsoft Corporation, CA, USA)^32^.

### Statistical analysis

Sample means were calculated for each variable and analysed for significance by one-way analysis of variance (ANOVA) followed by post-hoc testing with Dunnett’s method. Significance level, *α*, was set at 5% and hence differences were considered significant if *P* < 0.05.

## Results

### Suppression of CASP2 by siCASP2 results in RGC neuroprotection in vitro

**Fig. 1:**
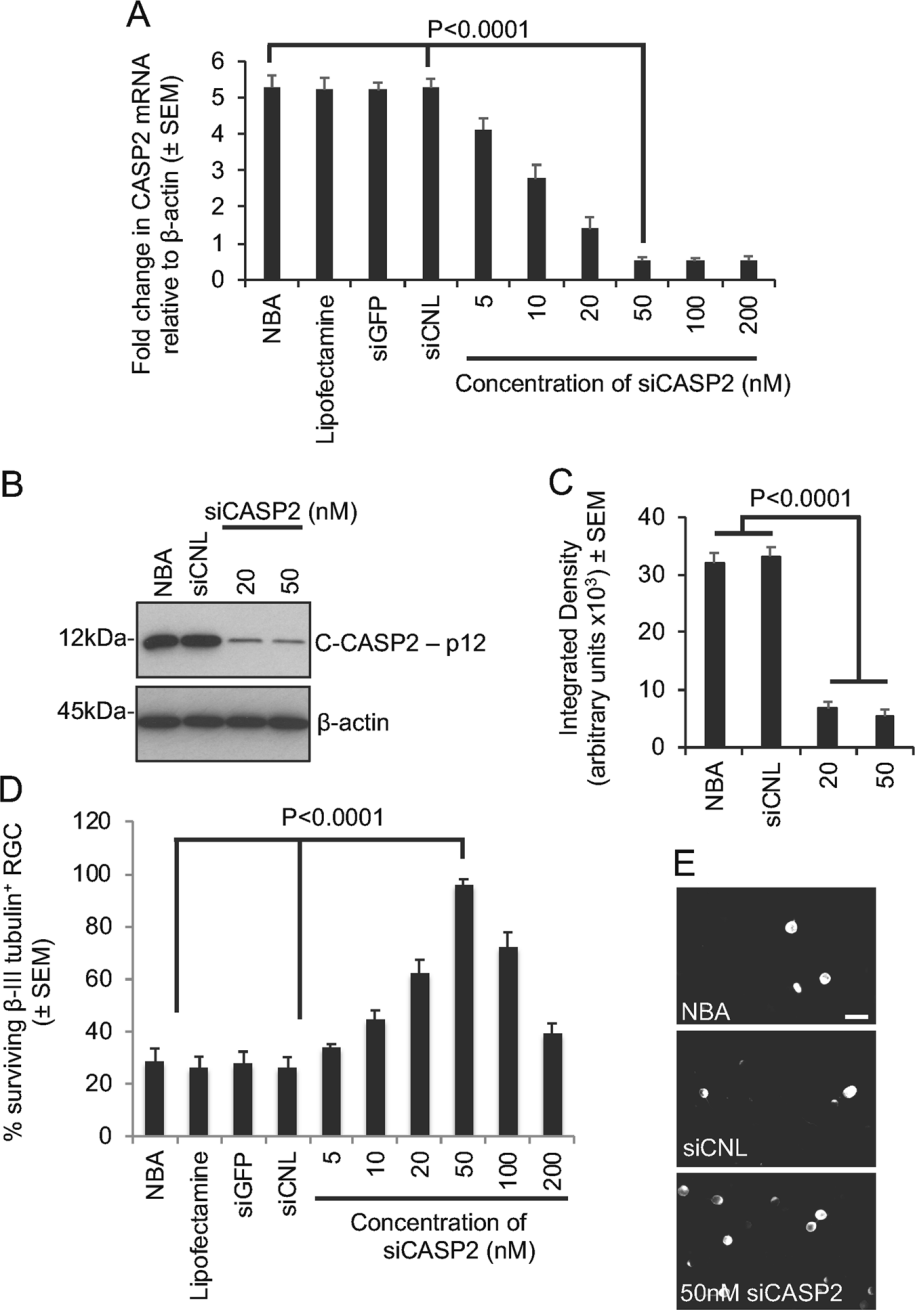
Validation of CASP2 knockdown by siCASP2 in vitro. **a** Fold change in CASP2 mRNA in siCASP2-treated retinal cells showing a dose-dependent decrease in CASP2, whilst Lipofectamine 2000 (Lipofectamine), non-specific siGFP and control scrambled siRNA to CASP2, siCNL, have no effect of CASP2 mRNA levels (*n* = 9 wells/treatment). **b**, **c** Western blot and subsequent densitometry to show significant reduction in C-CASP2 protein with 20 and 50 nM siCASP2 (*n* = 9 wells/treatment). **d** Quantification to show that knockdown of siCASP2 with an optimal dose of 50 nM promotes significant RGC neuroprotection in culture (*n* = 9 wells/treatment). **e** Representative images of selected groups to show morphology of βIII-tubulin^+^ RGC. Scale bar in **e** = 20 µm

In a preliminary experiment, we used siCASP2 to validate CASP2 mRNA knockdown in isolated adult rat retinal cultures containing RGC. In NBA-treated cultures, CASP2 mRNA was 5.3 ± 0.4-fold, which did not change in Lipofectamine, siGFP and siCNL-treated cultures (Fig. 1a). However, increasing the concentration of siCASP2 to 50 nM caused a dose-dependent decrease in CASP2 mRNA when only 0.53 ± 0.1-fold mRNA remained (Fig. 1a). Increasing the concentration of siCASP2 beyond 50 nM did not further reduce the levels of CASP2 mRNA. Western blot (Fig. 1b) and subsequent densitometry (Fig. 1c) was used to verify C-CASP2 protein knockdown by siCASP2 and showed that in NBA and siCNL-treated cultures high levels of C-CASP2 was activated whilst 20 and 50 nM siCASP2 significantly reduced C-CASP2 levels.

The percentage (%) of βIII-tubulin^+^ RGC in NBA, Lipofectamine, siGFP and siCNL-treated cultures were similar, showing that only 28 ± 5%, 27 ± 4% and 28 ± 4% RGC survived for 3d in culture, respectively (Fig. 1d, e). However, increasing the concentration of siCASP2 led to a dose-dependent increase in the % of surviving βIII-tubulin^+^ RGC, reaching a maximum of 95.6 ± 2.4% at 50 nM (Fig. 1d, e). Increasing the dose of siCASP2 above 50 nM led to a reduction in the % of βIII-tubulin^+^ RGC.

Taken together, these results demonstrate that 50 nM siCASP2 optimally reduced CASP2 mRNA and protein levels in cultured retinal cells and neuroprotects >95% of cultured RGC.

### PEDF suppresses CASP2 mRNA in cultured retinal cells

**Fig. 2:**
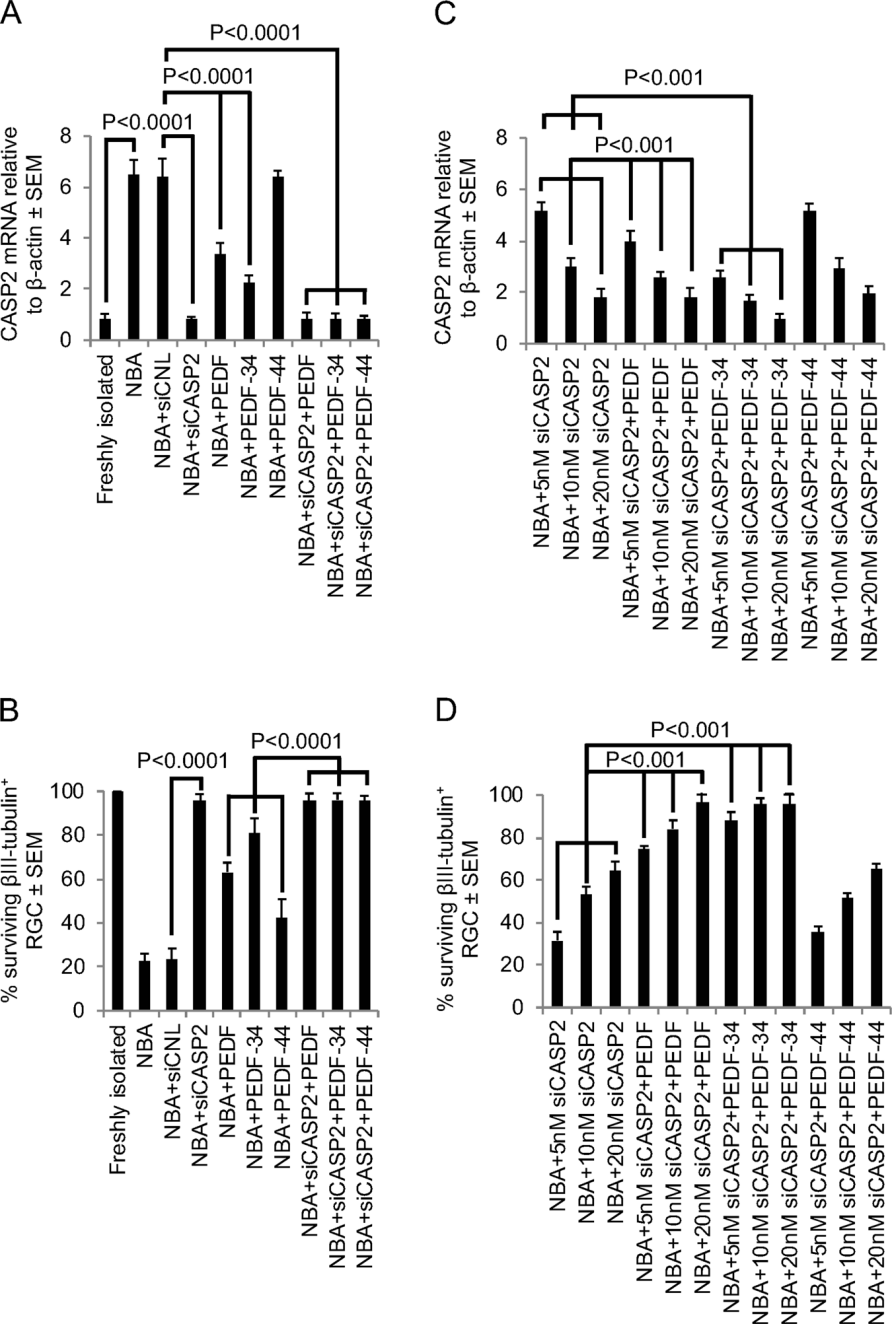
PEDF suppresses CASP2 and promotes neuroprotection in culture. **a** Fold change in CASP2 mRNA relative to β-actin reference gene in retinal cells freshly isolated and after 3d exposure to NBA, siCNL, 50 nM siCASP2 and PEDF/PEDF-34/PEDF-44. **b** % of surviving βIII-tubulin^+^ RGC after 3d in culture in NBA, and after treatment with siCNL, 50 nM siCASP2 and PEDF/PEDF-34/PEDF-44. **c** Fold change in CASP2 mRNA relative to β-actin reference gene in retinal cells freshly isolated and after 3d exposure to suboptimal doses of siCASP2 (5–20 nM) with and without PEDF/PEDF-34/PEDF-44. **d** % of surviving βIII-tubulin^+^ RGC after 3d in culture in with suboptimal concentrations of siCASP2 (5–20 nM) with and without PEDF/PEDF-34/PEDF-44 (*n* = 9 wells/treatment)

We showed previously that PEDF promoted significant RGC survival in vitro and in vivo and now speculated that PEDF may promote RGC neuroprotection through suppression of CASP2. Retinal cell cultures grown in NBA for 3d showed a 6.5 ± 0.58-fold increase in the levels of CASP2 mRNA compared to that observed in freshly isolated retinal cells (Fig. 2a). The rise in CASP2 mRNA levels in NBA and NBA + siCNL-treated cultures were suppressed completely by 50 nM siCASP2 (7.64-fold suppressed compared to NBA or NBA + siCNL-treated cultures), whilst PEDF and PEDF-34 also reduced the fold change in CASP2 mRNA to 3.36 ± 0.44 and 2.15 ± 0.33, respectively (Fig. 2a). These results equate to a significant 1.93- and 3.02-fold reduction in CASP2 mRNA after treatment with PEDF and PEDF-34, respectively, compared to cells grown in NBA or NBA + siCNL. PEDF-44, however, was unable to suppress CASP2 mRNA compared to NBA and NBA + siCNL-treated controls (Fig. 2a).

We confirmed that suppression of CASP2 mRNA by 50 nM siCASP2 and PEDF/PEDF-34 was neuroprotective to βIII-tubulin^+^ RGC. As a control for RGC neuroprotection, we compared neuroprotection offered by PEDF and PEDF-34 to that offered by 50 nM siCASP2. In NBA or NBA + siCNL-treated cultures, only 20.5 ± 4.6% of RGC survived for 3d (Fig. 2b). As expected, in all groups treated with 50 nM siCASP2, 95.6 ± 3.2% of RGC were protected from death, whilst PEDF and PEDF-34 promoted the survival of 63.2 ± 4.2% and 81.0 ± 6.9% of RGC from death, respectively. PEDF-44 had a smaller positive effect on RGC survival than either PEDF or PEDF-34, neuroprotecting 42.7 ± 8.3% of RGC. These results suggest that treatment of retinal cells with PEDF and PEDF-34 significantly suppress CASP2 mRNA levels and that RGC neuroprotection is proportional to suppressed CASP2 levels.

To test whether suppression of CASP2 mRNA observed with PEDF is additive when combined with suboptimal concentrations of siCASP2, we treated retinal cells with 5, 10 and 20 nM siCASP2, with or without PEDF. The combination of suboptimal concentrations of siCASP2 (i.e. 5, 10 and 20 nM) along with PEDF and PEDF-34 suppressed CASP2 mRNA levels in a dose-dependent manner, with PEDF-34 causing the greatest decrease (Fig. 2c). The level of suppression of CASP2 mRNA correlated directly with RGC neuroprotection, with NBA + 20 nM siCASP2 + PEDF-34 showing nearly 95% RGC neuroprotection (Fig. 2d). In contrast, PEDF-44 had no additive effects on CASP2 mRNA suppression (Fig. 2c) nor RGC neuroprotection (Fig. 2d) when combined with suboptimal siCASP2 concentrations.

Other preoptimized rat CASP2 siRNA sequence (S1-3), despite having different abilities to knock down CASP2 mRNA (Supplementary Fig. 1A) and promote RGC survival (Supplementary Fig. 1B) on their own were also similarly additive in the presence of PEDF and PEDF-34 as siCASP2.

Taken together, these results suggest that treatment of retinal cells with PEDF and PEDF-34 significantly suppress CASP2 mRNA levels and that RGC neuroprotection is proportional to suppressed CASP2 levels.

### PEDF suppresses CASP2 mRNA in vivo

**Fig. 3:**
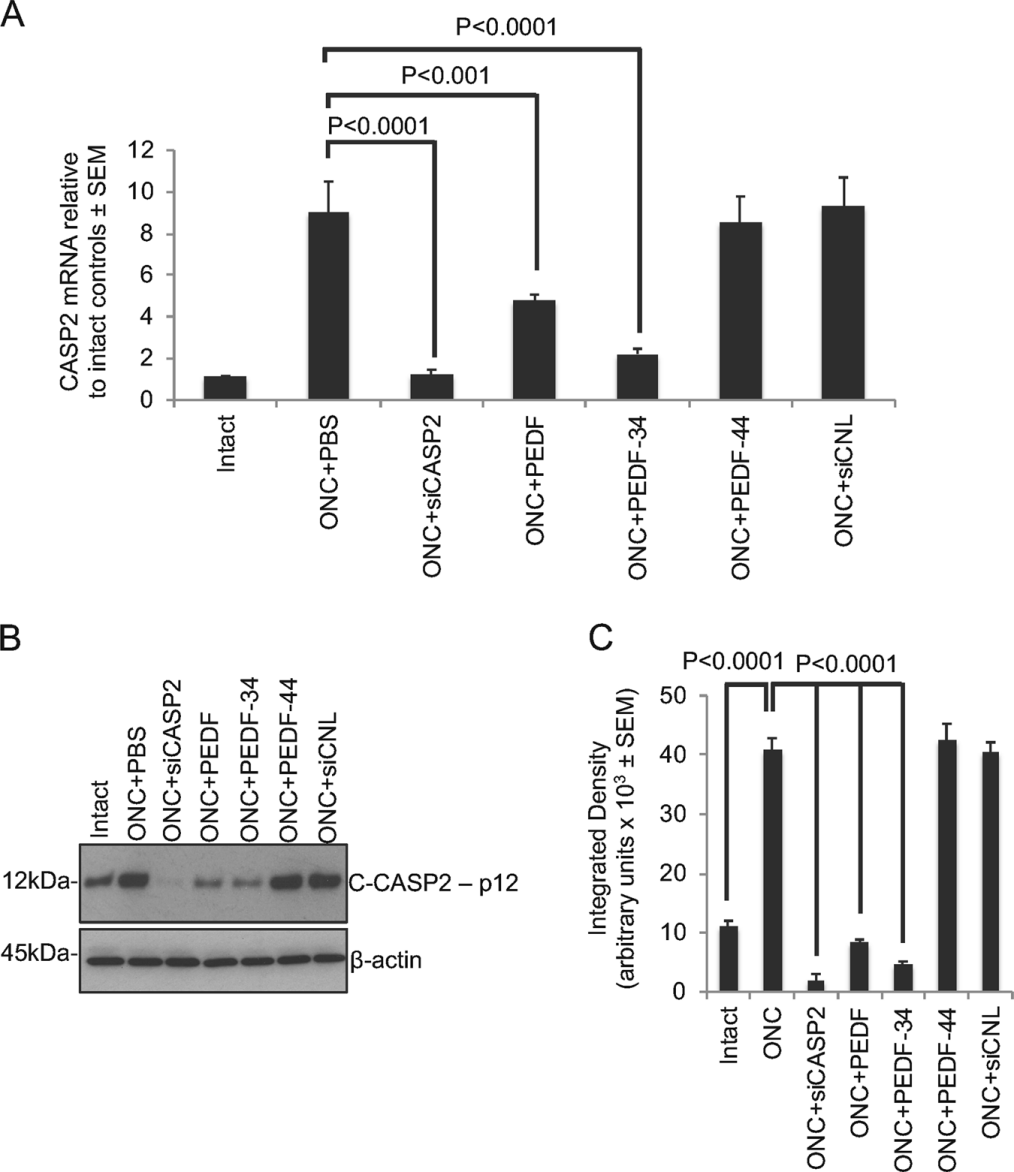
PEDF suppresses CASP2 in vivo. **a** Fold change in CASP2 mRNA at 7d after ONC + PBS, ONC + siCNL and ONC + siCASP2 and ONC + PEDF treatment. **b, c** Western blot and subsequent densitometry to show that ONC-induced C-CASP2 (p12 fragment) was significantly suppressed by siCASP2 and PEDF treatment (*n* = 18 retinae/treatment)

CASP2 mRNA levels rose to 8.63 ± 0.41-fold at 7d after ONC + PBS treatment compared to that observed in intact controls (Fig. 3a). Intravitreal injection of siCASP2 completely suppressed the ONC-induced rise in CASP2 mRNA such that levels were 1.24 ± 0.21 compared to intact controls. Intravitreal injection of PEDF and eye drops of PEDF-34 both significantly reduced ONC-induced CASP2 mRNA such that CASP2 mRNA levels were 4.73 ± 0.33 and 2.22 ± 0.23 compared to intact controls, a reduction of 1.82- and 3.89-fold compared to ONC alone. PEDF-44 had little or no effect on CASP2 mRNA levels. In addition, intravitreal injection of control siCNL had no effect on ONC-induced induction of CASP2 mRNA. These changes were also confirmed by western blot analysis for cleaved caspase-2 (C-CASP2) with antibodies specific for the p12 fragment showing that ONC-induced C-CASP2 protein levels were almost completely suppressed by siCASP2 and significantly reduced by PEDF and PEDF-34 (Fig. 3b, c). PEDF-44 and control siCNL had no effect on C-CASP2 levels compared to ONC + PBS-treated animals.

In addition, despite significant activation of cleaved caspase-3 (C-CASP3) and cleaved caspase-6 (C-CASP6) after ONC, levels of these and other executioner caspases including cleaved caspase-7 (C-CASP7) and cleaved caspase-8 (C-CASP8) all remained unchanged after siCASP2 and PEDF/PEDF-34 treatment (Supplementary Fig. 2). Taken together, these results suggest that only CASP2 mRNA and its active levels are significantly suppressed in vivo by siCASP2 and PEDF treatment.

### PEDF-34 eye-drops also suppressed CASP2 levels in vivo

**Fig. 4:**
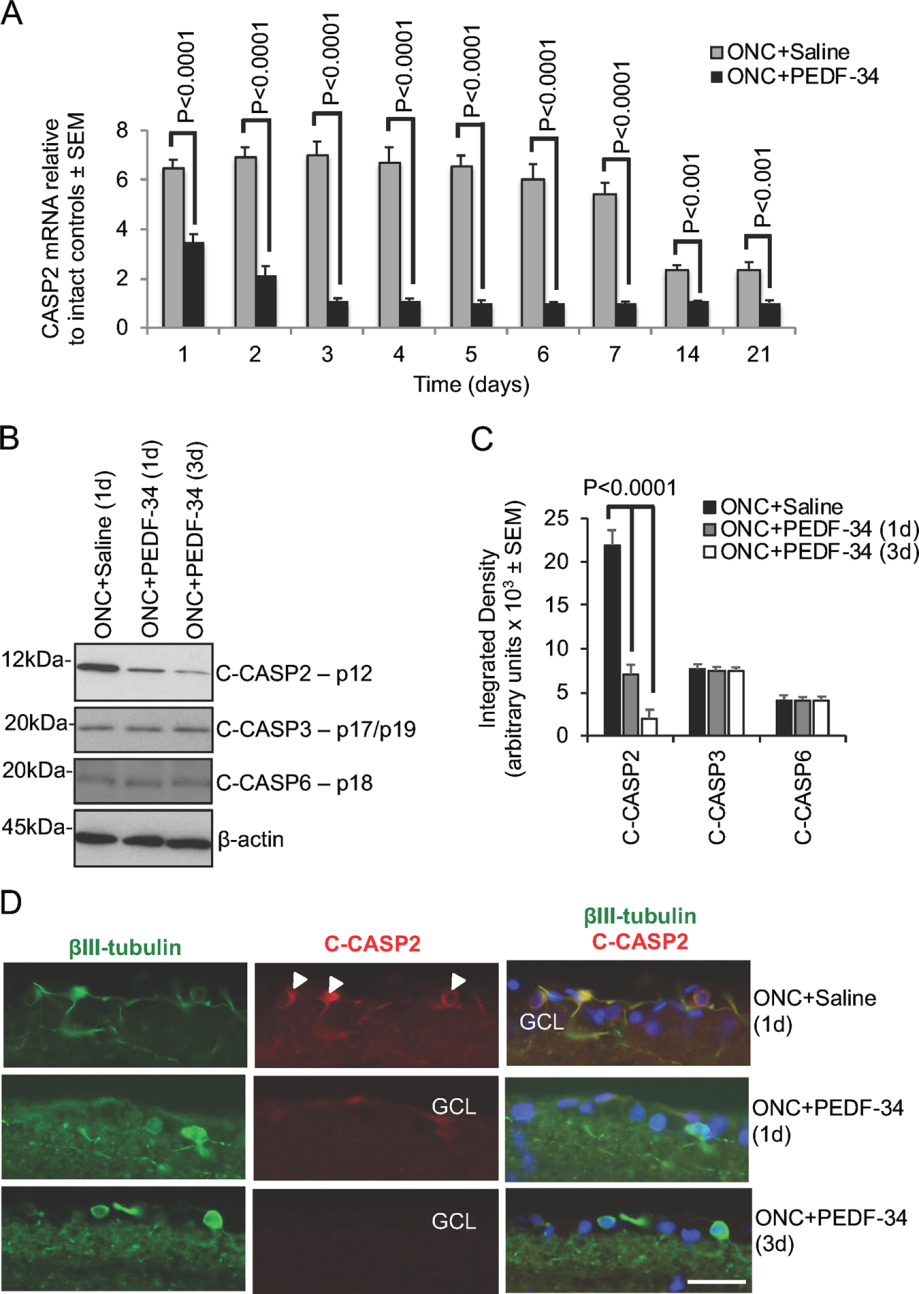
PEDF-34 eye-drops suppress CASP2 in retinae. **a** Fold change in CASP2 mRNA after ONC + saline and ONC + PEDF-34 daily eye-drops over 21d. **b**, **c** Western blot and subsequent densitometry to show that ONC-induced rise in C-CASP2 (p12 fragment) is significantly suppressed at 1d and 3d after injury and treatment, without changes in C-CASP3 or C-CASP6 levels. **d** Immunohistochemistry to show that ONC-induced localisation of C-CASP2 (red) in βIII-tubulin^+^ (green) RGC (arrowheads) after ONC + saline treatment is suppressed by 1d and 3d after ONC + PEDF-34 eye-drops. Scale bar = 50 µm. GCL = ganglion cell layer. (n = 18 retinae/treatment)

ONC + saline induced upregulation of CASP2 mRNA within 1d to 6.44 ± 0.38-fold over intact controls, peaking at 3d to 7.01 ± 0.54-fold and remained high for up to 7d. At 14 and 21d after ONC + saline, CASP2 mRNA remained at 2.33 ± 0.33-fold above that of intact controls (Fig. 4a). However, PEDF-34 eye-drops suppressed CASP2 mRNA 1d after ONC to 3.47 ± 0.37-fold, reducing further at 3d when CASP2 mRNA levels were 1.11 ± 0.13-fold above that observed in intact controls. No further reduction of CASP2 mRNA was observed after 3d, stabilising at approximately 7-fold less than that observed after ONC + saline treatment.

CASP2 western blots and subsequent densitometry reflected these changes and demonstrated significant reduction of C-CASP2 at 1d and 3d after ONC + PEDF-34 eye drop treatment compared to ONC + saline, without affecting C-CASP3 and C-CASP6 levels (Fig. 4b, c). Immunohistochemistry also showed that ONC-induced C-CASP2 immunoreactivity was reduced in βIII-tubulin^+^ RGC, disappearing at 1d and 3d after ONC + PEDF-34 eye-drop treatment (Fig. 4d). Taken together, these results suggest that eye drops of PEDF-34 suppressed ONC-induced CASP2 in the eye without affecting other executioner caspases.

### Glia contribute partially to the neuroprotective effects of PEDF

In mixed retinal cultures, where glial cell proliferation is inhibited with 5-FDU (Supplementary Fig. 3), siCASP2, PEDF and PEDF-34 treatment resulted in similar levels of CASP2 mRNA reduction as observed with mixed adult retinal cultures alone (Fig. 5a). In addition, the % of βIII-tubulin^+^ RGC reduced from 63 ± 4% and 82 ± 5% in mixed retinal cultures (compare with Figs. 2b) to 48 ± 2% and 65 ± 5% in 5-FDU-treated mixed retinal cultures after treatment with PEDF and PEDF-34, respectively (Fig. 5b).

**Fig. 5:**
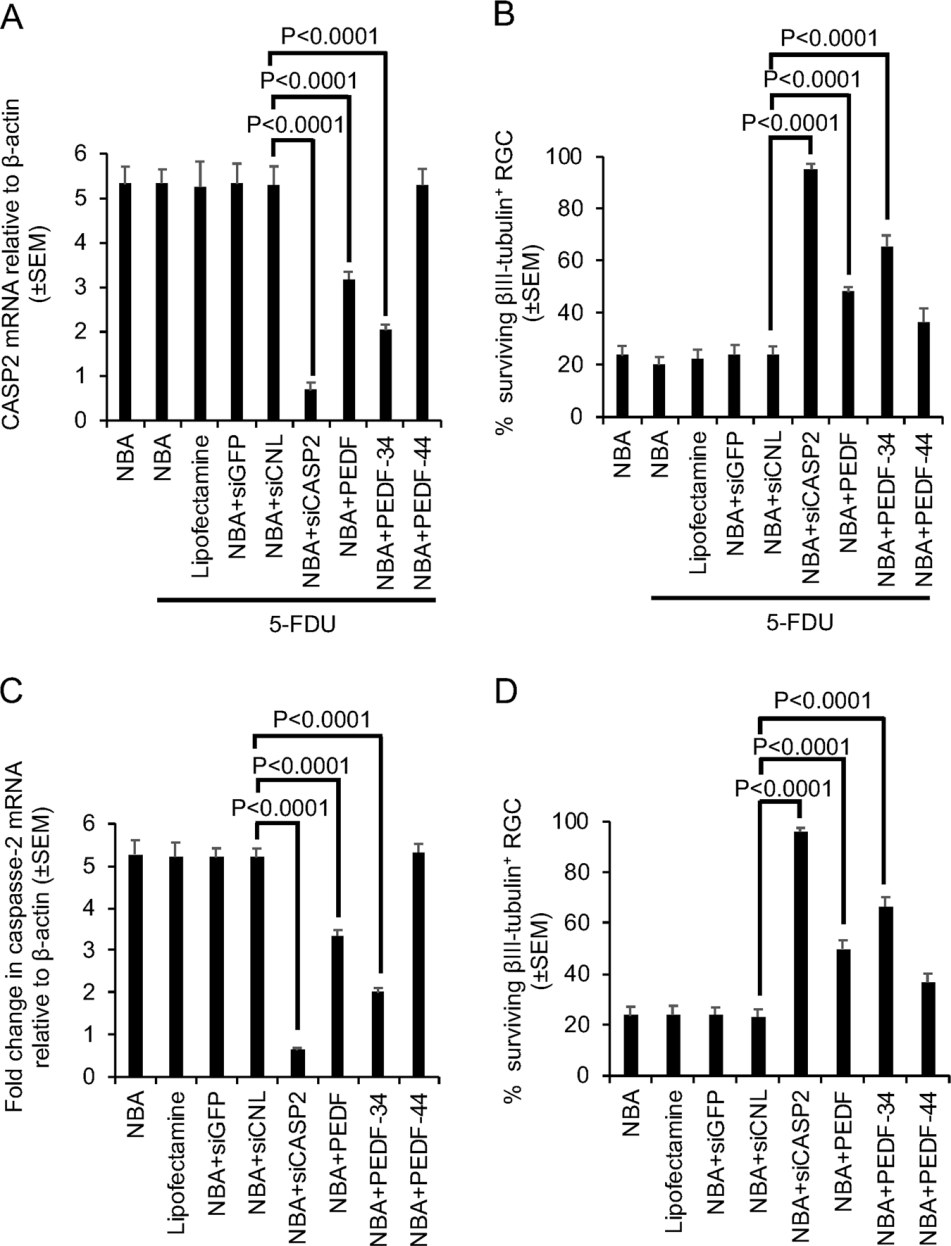
Glial cells contribute to some of the RGC neuroprotective properties of PEDF. **a** Fold change in CASP2 mRNA in 5-FDU-treated cultures showing no change in CASP2 mRNA after siCASP2 or PEDF treatment compared to cultures with glia. **b** % of surviving βIII-tubulin^+^ RGC in 5-FDU treated cultures in siCASP2 treated cells was unaffected but survival was reduced in the absence of glia. **c** Fold change in CASP2 mRNA in purified RGC cultures showing no change in CASP2 mRNA after siCASP2 or PEDF treatment. **d** % of surviving βIII-tubulin^+^ RGC in purified RGC cultures in siCASP2 treated cells was unaffected but survival was reduced in the absence of glia (*n* = 9 wells/treatment)

To confirm these effects, purified adult RGC cultures subjected to siCASP2, PEDF and PEDF-34 treatment, resulted in similar levels of CASP2 mRNA reduction as observed with mixed adult retinal cultures (Fig. 5c). The % of βIII-tubulin^+^ RGC did not differ after siCASP2 treatment when compared with mixed retinal cultures (Fig. 5c). However, the % of βIII-tubulin^+^ RGC reduced from 63 ± 4% and 82 ± 5% in mixed retinal cultures (compare with Fig. 2b) to 46 ± 3% and 64 ± 4% in purified RGC cultures after treatment with PEDF and PEDF-34, respectively (Fig. 5d). Taken together, these results show that PEDF suppresses CASP2 mRNA in in a glia-independent manner but exerts some of its neuroprotective effects through trans effects on glia.

### Retinal cells secrete NTFs in response to PEDF treatment which suppress C-CASP2 levels

**Fig. 6:**
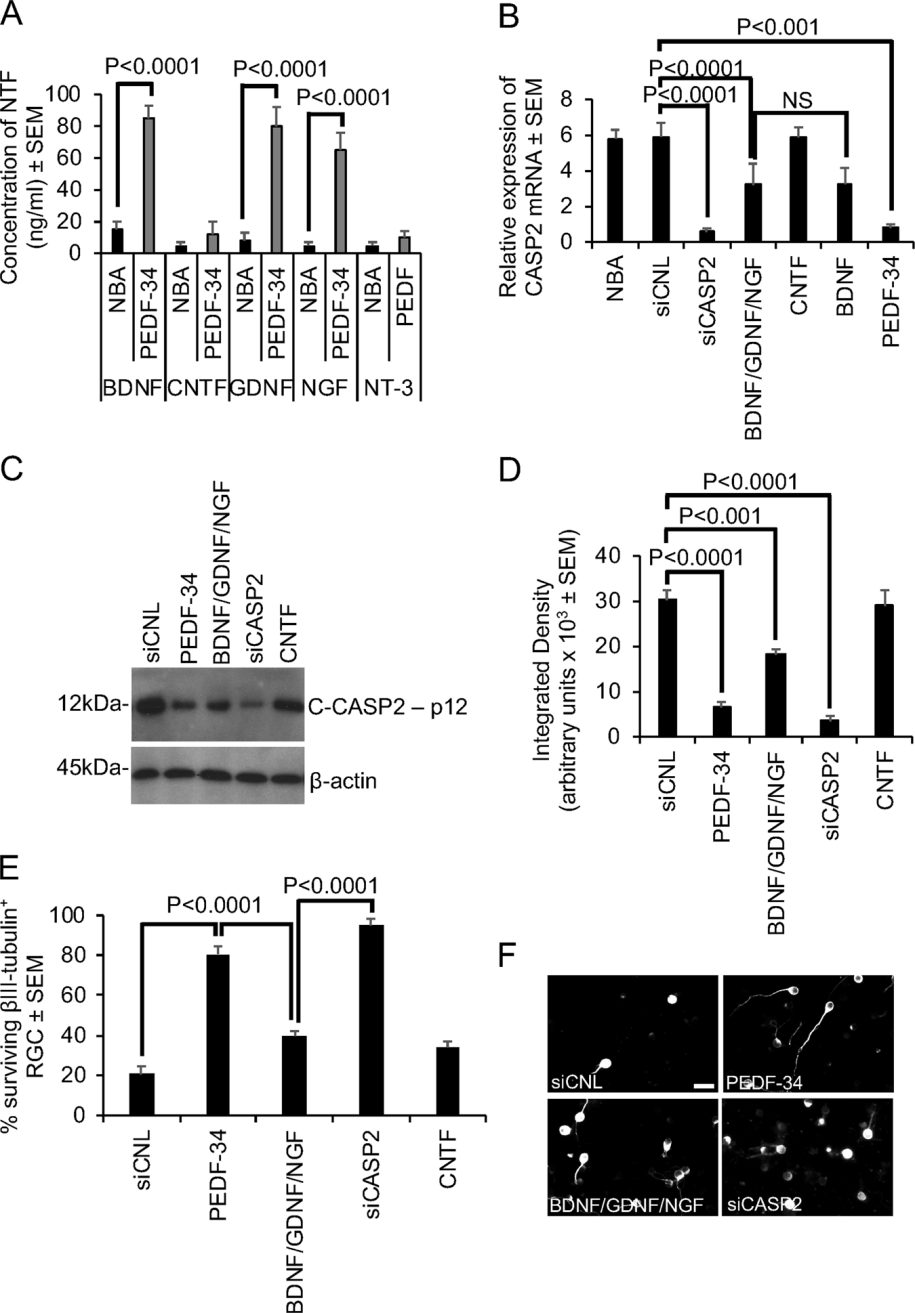
Treatment of retinal cultures with PEDF and PEDF-34 promotes the secretion of NTF. **a** ELISA to determine levels of BDNF, CNTF, GDNF, NGF and NT-3 released into the culture medium. Treatment of retinal cultures with ELISA isolated levels of BDNF, GDNF and NGF or CNTF either alone or in combination partially suppresses **b** CASP2 mRNA and C-CASP2 protein **c**, **d** by western blot and subsequent densitometry. **e** Representative images to demonstrate morphology of βIII-tubulin^+^ RGC (note: neurite outgrowth occurs in surviving βIII-tubulin^+^ RGC in wells containing PEDF, BDNF/GDNF/NGF and CNTF). (n = 9 wells/treatment). Scale bar in **f** = 20 µm

Mixed retinal cultures exposed to optimal concentrations of either PEDF (not shown) or PEDF-34 secreted 85 ± 8, 80 ± 12, 65 ± 11 ng/ml of BDNF, GDNF and NGF, respectively, into the culture medium (Fig. 6a). However, only baseline levels of CNTF and NT-3 were detected. These results suggest that mixed retinal cells exposed to PEDF-34 secrete significant titres of BDNF, GDNF and NGF.

We next determined if the secreted titres of BDNF, GDNF and NGF in combination can suppress CASP2 levels and thus account for the effects of PEDF-34 on CASP2. BDNF, GDNF and NGF in combination suppressed CASP2 mRNA by 45 ± 20% compared to NBA or siCNL-treated wells, whilst BDNF alone suppressed CASP2 mRNA levels by 44 ± 15% (Fig. 6b). CNTF had no effect on CASP2 mRNA but siCASP2 and PEDF-34 suppressed CASP2 mRNA levels by 90 ± 4 and 86 ± 5%, respectively, when compared to NBA or siCNL-treated well. Western blot (Fig. 6c) and subsequent densitometry (Fig. 6d) confirmed that: CNTF had no effect on C-CASP2 levels; BDNF, GDNF and NGF in combination suppressed C-CASP2 levels by 50% and that siCASP2 and PEDF-34 caused the greatest levels of C-CASP2 suppression. The reduction in C-CASP2 levels by BDNF, GDNF and NGF in combination also led to similar 50% promotion of RGC survival compared to siCNL-treated control groups and was also 50% of that afforded by PEDF-34 treatment alone (Fig. 6e, f).

Taken together, these results suggest that mixed retinal cells exposed to PEDF-34 secrete significant titres of NTF and that some of the effects of PEDF on CASP2 suppression and RGC survival may be related to the combined effects of BDNF, GDNF and NGF.

### Blocking the activity of PEDF with a polyclonal antibody prevents CAPS2 suppression

**Fig. 7:**
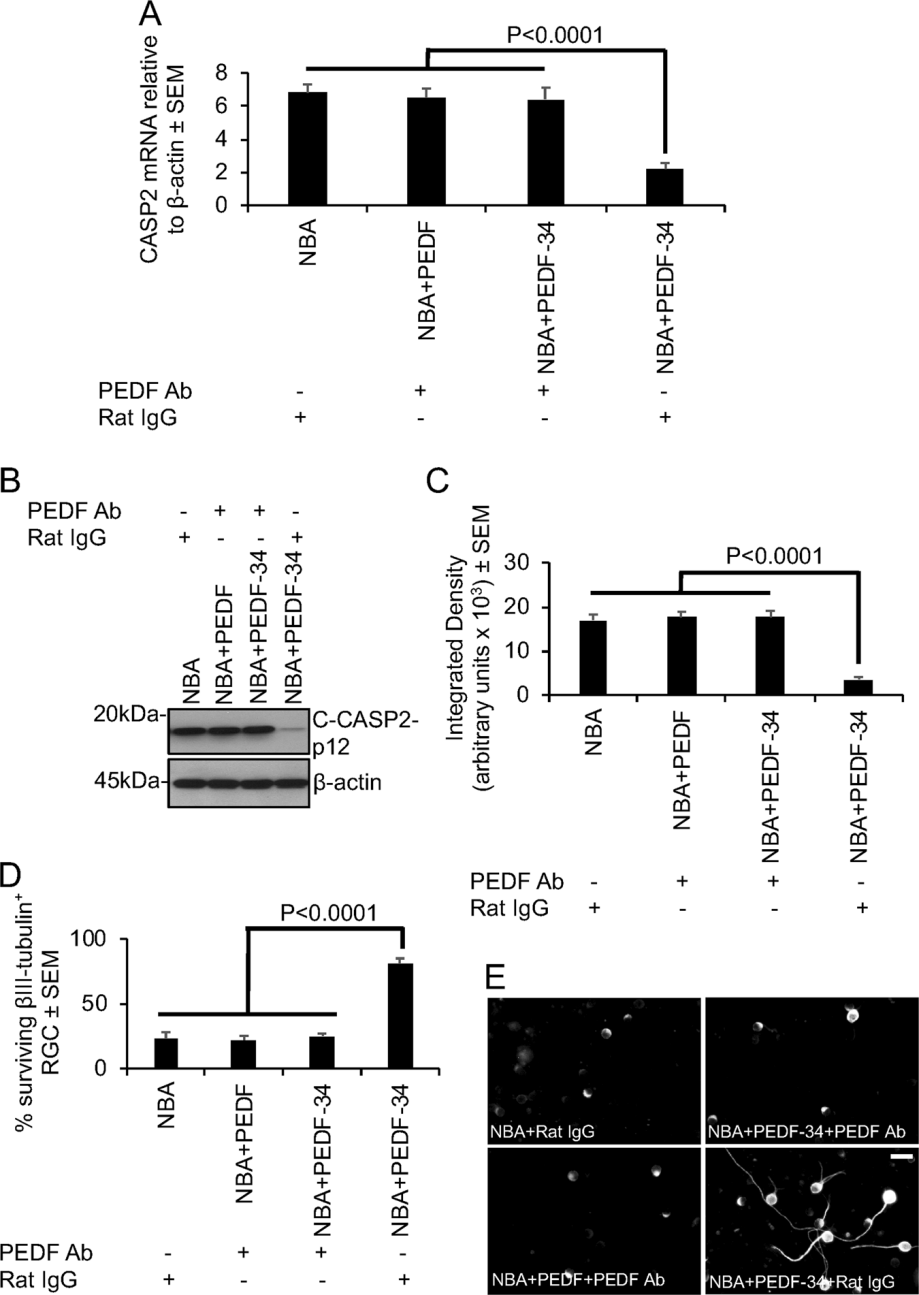
Antagonism of PEDF-34-mediated CASP2 mRNA and C-CASP2 protein reduction by pre-treatment with a polyclonal antibody to PEDF. **a**–**c** Pre-treatment of PEDF and PEDF-34 with a polyclonal antibody to PEDF fails to reduce CASP2 mRNA (**a**) and protein levels (**b**) whilst PEDF is suppressed in the absence of PEDF polyclonal antibody treatment, as confirmed by densitometry (**c**). **d** Pre-treatment with PEDF polyclonal antibody also suppresses the RGC neuroprotective effects of PEDF and PEDF-34. (n = 9 wells/treatment). **e** Representative images of the treatments in **d** to show morphology of βIII-tubulin^+^ RGC. Scale bar in **e** = 20 µm

We next determine if blocking the activity of PEDF can prevent its effects on CASP2 mRNA and protein suppression in mixed retinal cultures. In the presence of a polyclonal antibody to PEDF, neither PEDF nor PEDF-34 peptides were able to suppress CASP2 mRNA (Fig. 7a) or the levels of C-CASP2 protein (Fig. 7b, c). However, in the absence of the antibody to PEDF, PEDF-34 peptides significantly suppressed CASP2 mRNA (Fig. 7a) and C-CASP2 protein levels (Fig. 7b, c). Treatment of wells with the antibody to PEDF also blocked the neuroprotective effect of PEDF and PEDF-34 peptides on RGC survival, where similar levels of survival as control NBA-treated wells were observed (Fig. 7d, e). However, in the absence of the antibody to PEDF, the neuroprotective effect of PEDF-34 peptide was restored, leading to the survival of 81 ± 5% of RGC (Fig. 7d, e).

These results demonstrate that blocking the activity of PEDF, prevents its biological effects on CASP2 suppression and thus RGC neuroprotection.

### PEDF suppresses C-CASP2, RAIDD, downstream phospho-c-Jun and Bim

**Fig. 8:**
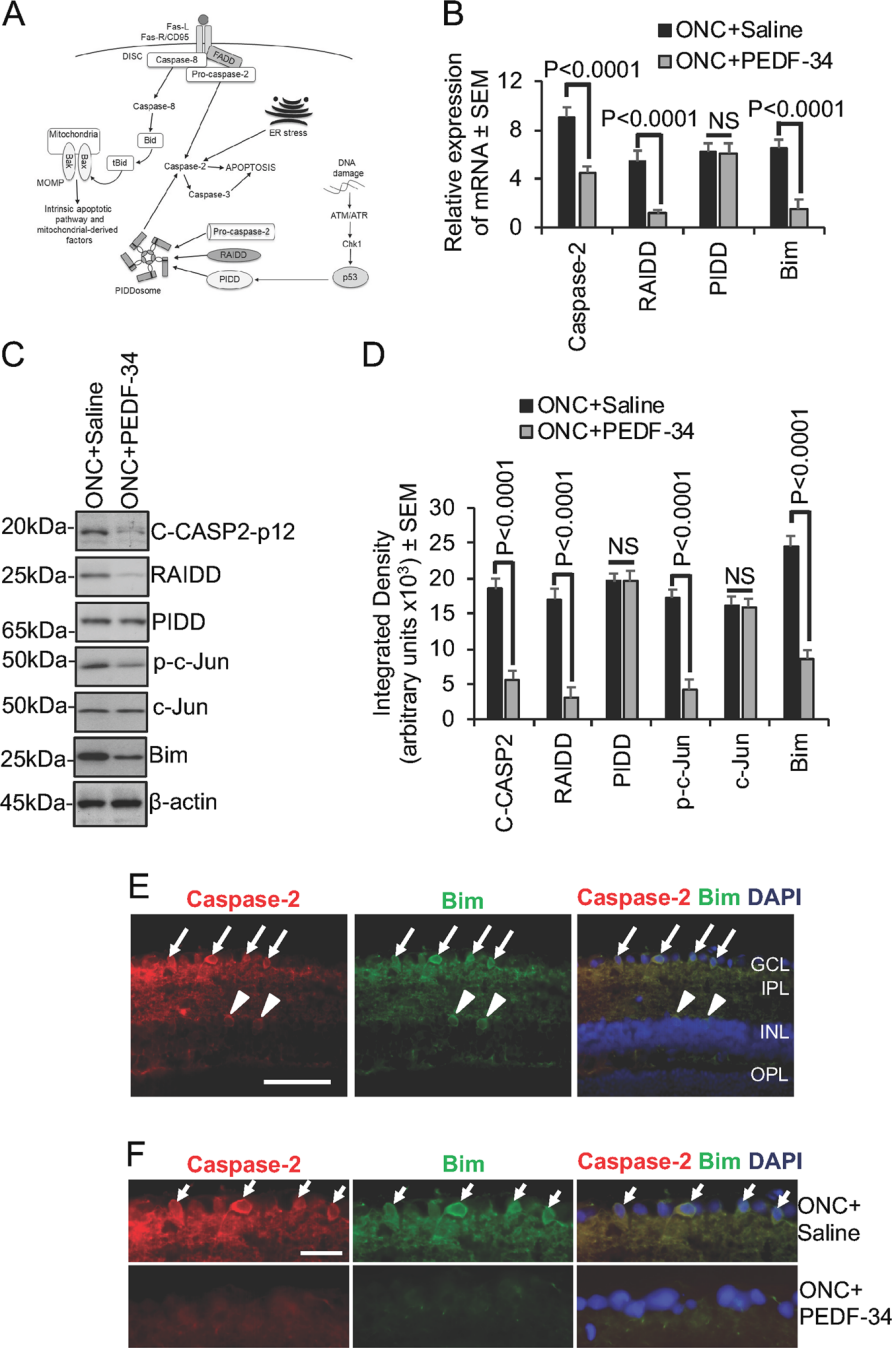
PEDF suppresses components in the CASP2-dependent apoptotic pathway after ONC in vivo. **a** CASP2-dependent apoptosis requires the formation of the PIDDosome (Adapted from^24^). **b** PEDF treatment suppresses CASP2, RAIDD but not PIDD and downstream Bim mRNA. **c**, **d** PEDF treatment suppresses C-CASP2, RAIDD, phopho-c-Jun and downstream Bim as detected by western blot and densitometry. **e**, **f** Immunohistochemistry to show changes in Bim protein colocalised to caspase-2 in the ganglion cell layer (GCL), inner plexiform layer (IPL), occasional cells of the inner nuclear layer (INL) whilst high power views of the GCL showed that Bim and caspase-2 were colocalised to RGC. Scale bars in **e** and (**f**) = 50 µm (*n* = 18 retinae/treatment)

To explore the mechanism of RGC death by CASP2 and the effect of CASP2 suppression by PEDF, we investigated components of the CASP2 activation and neuronal death pathway (Fig. 8a) in retinal mRNA and protein in the presence or absence of PEDF-34. We show that the PEDF-34 not only suppressed CASP2 mRNA (by 50%) but significantly suppressed RAIDD (by 80%) but not PIDD, and downstream Bim (by 77%) (Fig. 8b). Western blot (Fig. 8c) and subsequent densitometry (Fig. 8d) confirmed significant changes in C-CASP2, RAIDD but not PIDD and downstream Bim, whilst phospho-c-Jun was also significantly suppressed by PEDF. Immunohistochemistry for downstream Bim in retinal sections showed CASP2 and Bim^+^ immunoreactivity in the ganglion cell layer (arrows), inner plexiform layer, occasional cells in the inner nuclear layer (INL; arrowheads) and the outer plexiform layer (Fig. 8e). CASP2^+^ immunoreactivity was colocalised with Bim^+^ immunoreactivity in the ganglion cell layer corresponding to RGC (Fig. 8f; arrows).

Taken together, these results demonstrate that PEDF suppresses key components of the CASP2 activation pathway and thus promotes RGC neuroprotection.

## Discussion

In retinal cultures, we confirmed that siCASP2 caused > 95% RGC neuroprotection^20^ and that PEDF and PEDF-34 also exhibited significant and similar RGC neuroprotection as reported by us previously^11,12^. We also observed that the pre-optimised doses of PEDF and PEDF-34 in in vitro and in vivo caused significant and specific reduction in CASP2 mRNA and protein, without affecting other executioner caspases, and that this reduction in CASP2 was RGC neuroprotective. PEDF and PEDF-34 but not PEDF-44 were marginally additive in terms of CASP2 mRNA suppression when treated alongside suboptimal concentrations of siCASP2. In addition, some of the neuroprotective effects of PEDF were mediated by trans effects on glia. Blocking the activity of PEDF by pre-incubation with a polyclonal antibody abolished the neuroprotective effects of PEDF and PEDF-34 on RGC survival. We also show that suppression of CASP2 by PEDF also suppressed RAIDD and downstream phospho-c-Jun and Bim levels in the pathway to CASP2-dependent apoptosis in RGC.

We have previously shown that ONC specifically activates CASP2 in RGC and suppression of CASP2 with siCASP2 protected > 95% of RGC for up to 84d^11,22,23^. We have also shown that PEDF and PEDF-34 promote significant RGC neuroprotection and neurite outgrowth/axon regeneration in vitro and in vivo^11,12^. Our present study suggests that exogenous PEDF may offer RGC neuroprotection by specifically reducing CASP2. Exogenous PEDF has been shown to downregulate apoptotic genes such as CASP2, calpain-1 and MAPK-1 after binding to one or more of the high affinity PEDF receptors, thus stimulating phospholipase A2 activity^8,10^. Although these studies were performed using established cell lines such as in Y79 retinoblastoma cells and retinal pigment epithelium (RPE) cells^8,10^, the reduction in CASP2 after exogenous PEDF addition is in agreement with our current study in primary RGC in vitro and after ONC in vivo.

PEDF signalling has not yet been fully elucidated, but inhibition of both nuclear factor kappa-light-chain-enhancer of activated B cells (NFκB) and ERK1/2 pathways abolished the protective effects of PEDF on RGC in vitro^8,33,34^. In addition, PEDF-activated ERK1/2 modulates kinases, phosphatases, transcription factors and regulators of apoptosis, suggesting multiple pathway activation roles for PEDF^35,36^. For example, the activation of CASP2 in hippocampal neurons in response to NGF withdrawal or Aβ_1-42_ treatment, was shown to be dependent on the adaptor protein RAIDD but not PIDD and that both CASP2 and RAIDD were required for downstream c-Jun activation and Bim induction^27,28^. Our results agree with these studies and demonstrate that after ONC, high levels of RAIDD (but not PIDD), phopho-c-Jun and Bim were detected which were all suppressed by PEDF treatment suggesting that RGC undergo CASP2-dependent apoptosis through the c-Jun and Bim pathway.

Interestingly, treatment of retinal cultures with PEDF induced the production of high titres of BDNF, GDNF and NGF but not CNTF and NT3, all of which are RGC neuroprotective. Although low levels of CNTF were detected in response to PEDF treatment, CNTF and BDNF have similar effects on RGC survival after ONC^37,38^. BDNF administration promotes the survival of nearly 100% of RGC for 7 days^20^ but by 14d after ONC, only ~45% of RGC remain in BDNF-treated eyes^39^. Sustained RGC neuroprotection has not been observed with BDNF since its high affinity receptor, TrKB, is rapidly downregulated in response to ON injury^40–42^. In addition, BDNF diminishes CASP2 but not c-Jun immunoreactivity and this reduction in CASP2 may account for some of its neuroprotective effects on RGC. In contrast, PEDF not only suppressed CASP2 but also RAIDD and downstream phospho-c-Jun and Bim, suggesting that PEDF may have a different mode of action compared to BDNF. GDNF is neuroprotective for RGC survival and axon regeneration but at 14 days after ONC, only 50% of RGC remained in GDNF-treated eyes^43–47^. GDNF gene transfer to axotomized RGC also resulted in RGC survival for 2-4 weeks with a concomitant decrease in caspase-3 and -9^48^. NGF is also RGC neuroprotective but at 5 weeks after ON injury, <4% of RGC survived despite repeated intraocular injections^49^. Given the neuroprotective properties of all of these NTF on RGC, it is conceivable that these NTF work in concert with PEDF to produce the overall RGC neuroprotection observed by us after PEDF treatment.

PEDF is normally expressed in pigment, ciliary, corneal epithelial cells, Muller cells and in astrocytes and RGC. In addition, retinal-glia-derived PEDF is increased after exposure to exogenous PEDF^11^ and therefore may augment endogenous levels of PEDF, contributing to the overall neuroprotective effect of PEDF. PEDF compares well to other neuroprotective agents such as BDNF, GDNF, NT-3 and TrkB gene transfer, and to caspase inhibitors. Like BDNF, PEDF may suppress CASP2, accounting for RGC neuroprotection. However, some of the effects of PEDF on RGC neuroprotection were due to trans effects on glia since 5-FDU treated and purified RGC cultures showed reduced levels of RGC neuroprotection. It may be that despite constitutive expression of PEDF in the retina, intravitreal injection or eye drops of PEDF may stimulate autocrine-induced PEDF production in RGC, astrocytes and Muller cells^21^. Therefore, secreted glia-derived PEDF may also supplement exogenous PEDF titres to augment RGC neuroprotection. PEDF is also likely to function both directly and indirectly by suppressing CASP2 activity and as a consequence preserving glutamine synthetase levels, preventing the accumulation of reactive oxygen species and counteracting the detrimental effects of gliosis^11,50^.

The clinical significance of this work is that PEDF and PEDF-34 are not only RGC neuroprotective, but also promote their axons to regenerate and hence represents a single molecule that could be beneficial in the fight against vision loss. In addition, PEDF-34 mimics the activity of full length PEDF but can be delivered through eye drops, making it an attractive molecule for therapy since it reduces the need for intravitreal injections and thus alleviates pain and the risk of infection. In contrast, siCASP2 is remarkably neuroprotective but does not promote RGC axon regeneration and hence may require additional axon regenerative stimuli such as PEDF. Despite this observation, siCASP2 is currently in Phase II and III clinical trials for use in non-arteritic anterior ischaemic optic neuropathy and acute primary angle closure glaucoma, respectively.

In conclusion, we confirm our previous reports that PEDF is RGC neuroprotective and demonstrate that this neuroprotective effect may be mediated by suppression of CASP2. These findings re-affirm the useful role of PEDF as a potential therapeutic in mediating RGC viability during the treatment of optic neuropathies in which RGCs die.

## Supplementary information

Supplementary Figure 1

Supplementary Figure 2

Supplementary Figure 3

Supplemental figure legends
