# Supplementary figures and images for: Pigment epithelium-derived factor mediates retinal ganglion cell neuroprotection by suppression of caspase-2
Source: Cell Death Dis. 2019 Feb 4;10(2):102. doi: 10.1038/s41419-019-1379-6 (PMC6362048; doi:10.1038/s41419-019-1379-6)

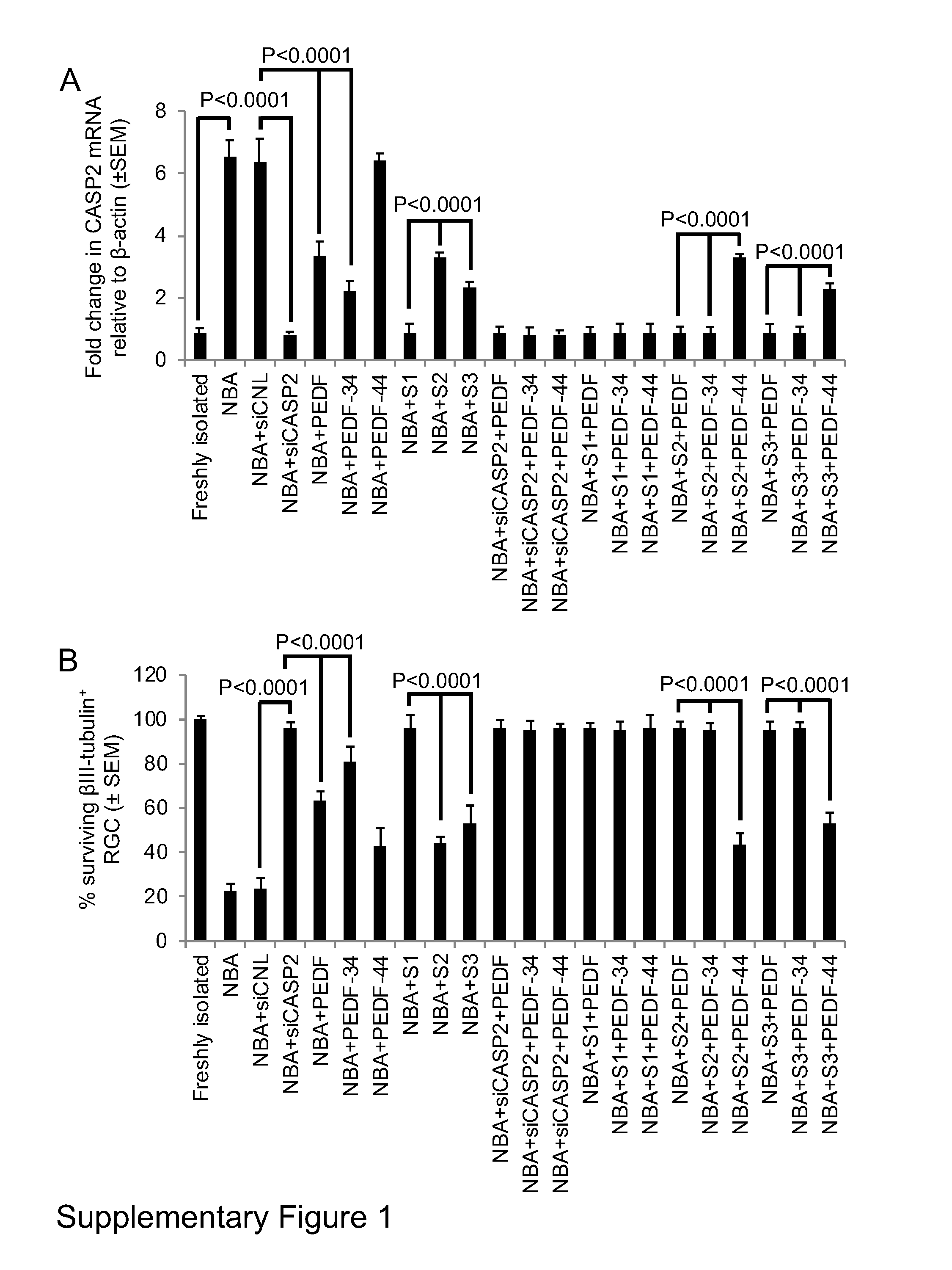

Supplement: Supplementary file 1 — Supplementary Figure 1 [file 41419_2019_1379_MOESM1_ESM.tif]

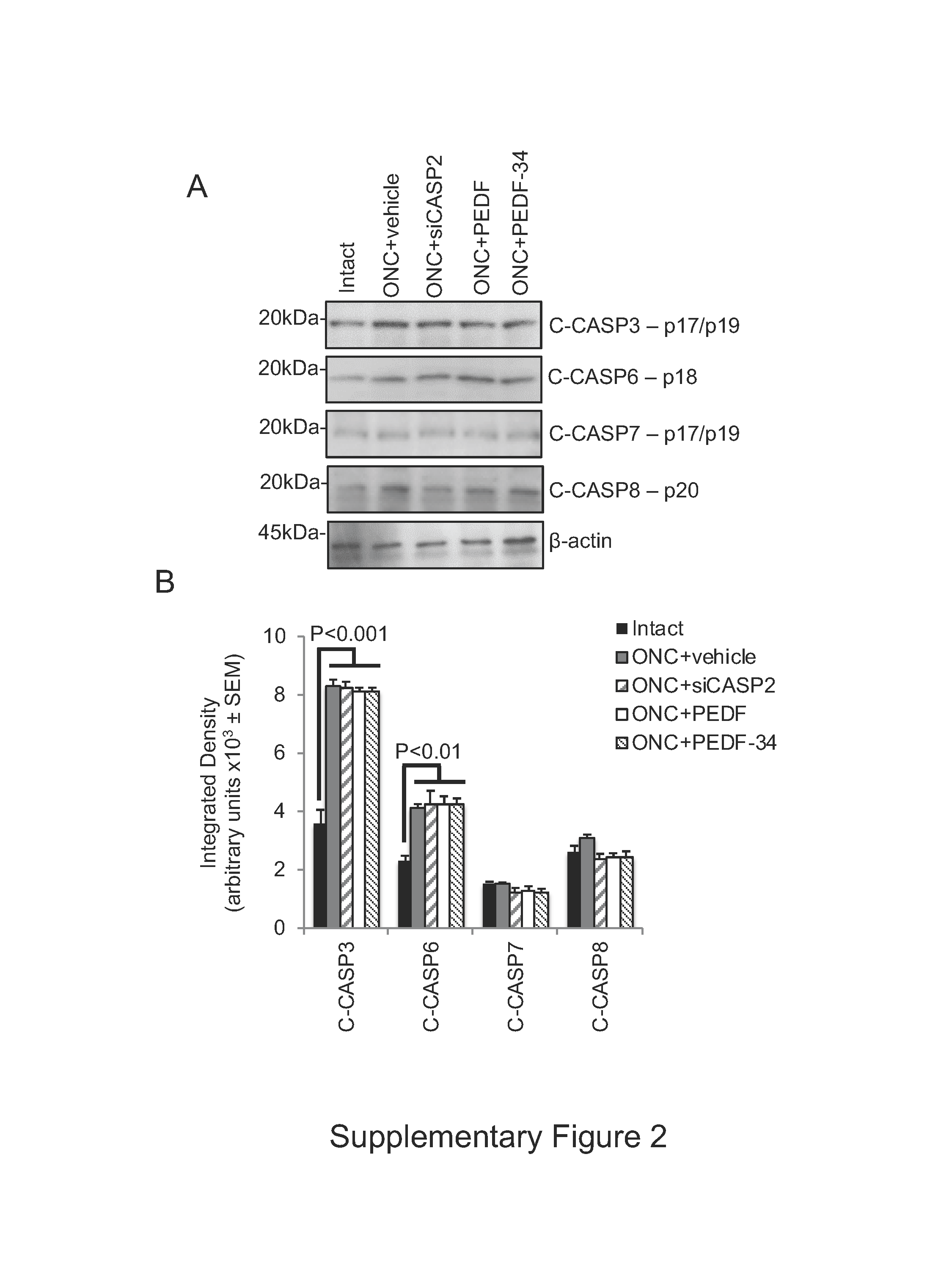

Supplement: Supplementary file 2 — Supplementary Figure 2 [file 41419_2019_1379_MOESM2_ESM.tif]

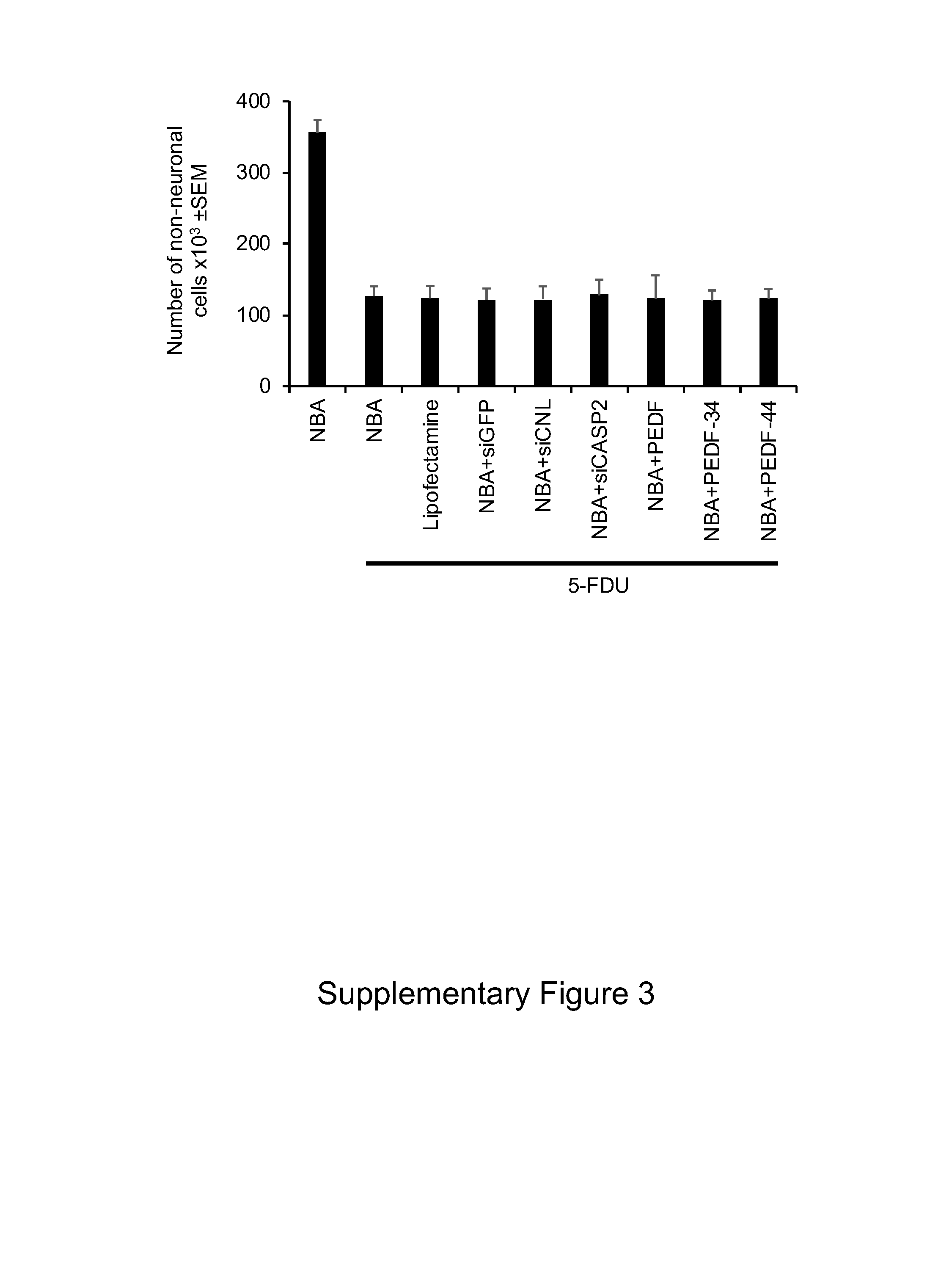

Supplement: Supplementary file 3 — Supplementary Figure 3 [file 41419_2019_1379_MOESM3_ESM.tif]
