# Supplementary material for: Pigment epithelium-derived factor mediates retinal ganglion cell neuroprotection by suppression of caspase-2
Source: Cell Death Dis. 2019 Feb 4;10(2):102. doi: 10.1038/s41419-019-1379-6 (PMC6362048; doi:10.1038/s41419-019-1379-6)
Supplement: Supplementary file 4 — Supplemental figure legends [file 41419_2019_1379_MOESM4_ESM.docx]

**Supplementary Figures**

**Supplementary Figure 1.** PEDF suppresses CASP2 and promotes neuroprotection in culture. (**A**) Fold change in CASP2 mRNA relative to β-actin reference gene in retinal cells freshly isolated and after 3d exposure to NBA, siCNL, 50nM siCASP2, PEDF/PEDF-34/PEDF-44 and 50nM S1, S2 and S2 (all rat siRNA to CASP2) +/-PEDF. (**B**) % of surviving βIII-tubulin^+^ RGC after 3d in culture in NBA, after treatment with siCNL, 50nM siCASP2 and PEDF/PEDF-34/PEDF-44 and 50nM S1, S2 and S2 +/-PEDF.

**Supplementary Figure 2.** No changes in other executioner caspases after treatment with either siCASP2 or PEDF. **a, b** Western blot and subsequent densitometry shows activation of C-CASP-3 and C-CASP6 after ONC but no changes were detected in C-CASP3, C-CASP6, C-CASP7 or C-CASP8 after ONC plus treatment with siCASP2 or PEDF/PEDF-34. β-actin is used as a loading control. (n = 18 retinae/treatment).

**Supplementary Figure 3.** Quantification of glial cells in retinal cultures treated with 5-FDU showing greater than 2-fold reduction glial cells in 5-FDU treated cultures. (n = 9 wells/treatment).
